# Prominent corneal nerves in pure mucosal neuroma syndrome, a clinical phenotype distinct from multiple endocrine neoplasia type 2B

**DOI:** 10.1186/s12886-023-03005-0

**Published:** 2023-06-12

**Authors:** L Yin, YNZ Wang, J Zhu, CY Tan, C Sun, Y Yao

**Affiliations:** Department of Ophthalmology, Wuxi People’s Hospital Affiliated of Nanjing Medical University, Qingyang Road 299, Wuxi, 214002 China

**Keywords:** Prominent corneal nerves, In vivo confocal microscopy (IVCM), Neurofibromatosis, Pure mucosal neuroma syndrome (MNS)

## Abstract

**Background:**

Pure mucosal neuroma syndrome (MNS), an autosomal dominant neurocutaneous disorder, is a rare discrete subgroup in multiple endocrine neoplasia (MEN) type 2B, which present without associated endocrinopathies of MEN2B but with typical physical features such as prominent corneal nerves.

Case presentation

This report describes a 41-year-old patient with complaint of itchy eyes and irritation, presenting with blocked gland orifices in the upper and lower eyelids, light conjunctival hyperemia, a semitransparent neoplasm measuring 2 mm*2 mm on the nasal limbus suggestive of neuromas, and prominent corneal nerves. In vivo confocal microscopy (IVCM) revealed structural alterations—namely a prominent hyperreflective, thickened nerve plexus and a normal endothelium—in both eyes. Testing for SOS1 mutation was positive. This patient may represent a discrete subgroup termed pure mucosal neuroma syndrome (MNS), which presents with the characteristic appearance of MEN2B but without RET gene mutations.

**Conclusion:**

Prominent corneal nerves have been described in some diseases, such as multiple endocrine neoplasia (MEN) type 1 and type 2A and 2B, congenital ichthyosis, Refsum’s disease, leprosy, etc. Ophthalmic assessment including prominent corneal nerves has proven valuable in asymptomatic individuals of MEN2B. Our case illustrates the importance of recognizing the ocular features of MNS, a rare presentation of MEN2B, in order to prevent prophylactic thyroidectomy in these patients for prophylactic thyroidectomy is not mandatory in MNS. However, regular monitoring and genetic counseling are still necessary.

## Background

The cornea is one of the most sensitive tissues in the human body. The cornea nerves and sensations originate from the nasociliary branch of the first ophthalmic division of the trigeminal nerves (cranial nerve V), forming a plexus at the corneoscleral limbus [1]. From the limbal plexus, unmyelinated nerve endings maintaining transparency terminate in the corneal epithelium [2, 3]. In the superficial cornea, the nerve plexus penetrates into the Bowman’s membrane and is distributed beneath the basal epithelial layer. Thickened corneal nerves are visible due to the myelination of corneal nerves [4].

Corneal nerves may be apparently thickened and more visible in some ocular diseases, such as Fuchs’ endothelial dystrophy, acanthamoeba keratitis, keratoconus, congenital glaucoma, herpes simplex, herpes zoster, posterior polymorphous dystrophy, and corneal transplantation failure [5–7]. Meanwhile, prominent corneal nerves are associated with systemic disorders such as Leprosy (Hansen's Disease), primary amyloidosis, pheochromocytoma, Refsum's disease, Marfan syndrome, congenital ichthyosis, ectodermal dysplasia, and neurofibromatosis. Neurofibromatosis, a rare autosomal dominant disorder, encompasses at least three distinct disorders, referred to as NF1, NF2, and schwannomatosis. NF1, known as Von Recklinghausen’s disease, is the most common type of neurofibromatosis, with an estimated incidence of approximately 1 in 3,000 individuals to 1 in 3,500 individuals worldwide [8–10]. The clinical manifestations of NF1 are extremely varied in expressivity. Two or more features are required to establish a diagnosis of NF1: six or more café-au-lait macules (> 5 mm in diameter in prepubertal individuals and > 15 mm after puberty), two or more neurofibromas or one plexiform neurofibroma, skin-fold freckling, two or more Lisch nodules, characteristic skeletal dysplasia (long bone or sphenoid wing), and a first-degree relative affected [11]. Of note, Lisch nodules, and plexiform neurofibromas are hallmarks and considered diagnostic for NF1.

Also, prominent corneal nerves are related to the topic of this case presentation, multiple endocrine neoplasia (MEN) including MEN type 1 and type 2A and 2B. Prominent corneal nerves are one of the characteristic phenotypic features of MEN2B, which can provide an early clue to the diagnosis of the syndrome. Rarely, patients present with typical physical features (mucosal neuromas, prominent corneal nerves and marfanoid body habitus) of MEN2B but without a RET gene mutation or associated endocrinopathies (medullary thyroid carcinoma or pheochromocytoma). Pure mucosal neuroma syndrome (MNS), an autosomal dominant neurocutaneous disorder, is a rare discrete subgroup in MEN2B [12], which presents without associated endocrinopathies of MEN2B but with typical physical features such as prominent corneal nerves.

## Case presentation

**Fig. 1:**
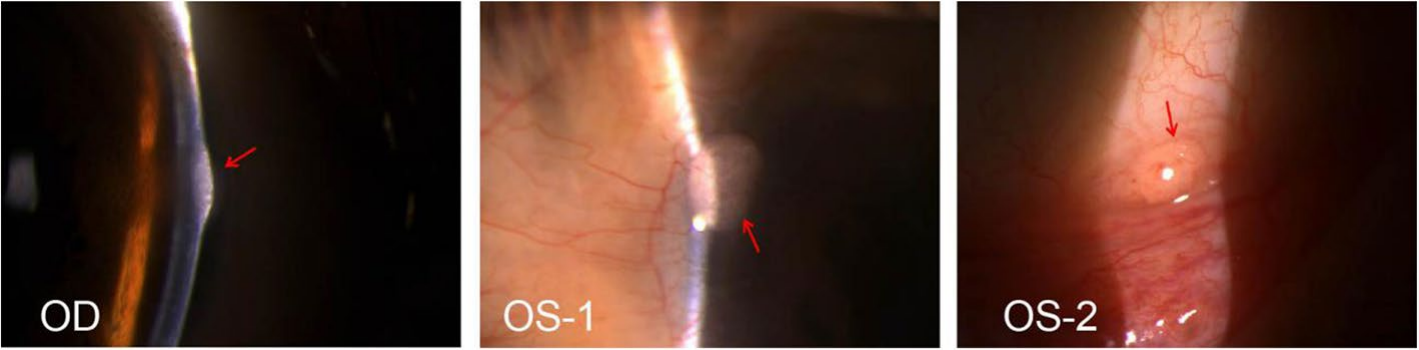
Slit-lamp images of semitranslucent neoplasm (OD and OS, red arrowhead). OS-1, Semitransparent neoplasm on nasal corneoscleral limbus of left eye, with ingrowing blood vessels, (2 mm*2 mm size). OS-2, Neoplasm on the inferior conjunctival fornix of left eye, (2 mm*1.5 mm size)

**Fig. 2:**
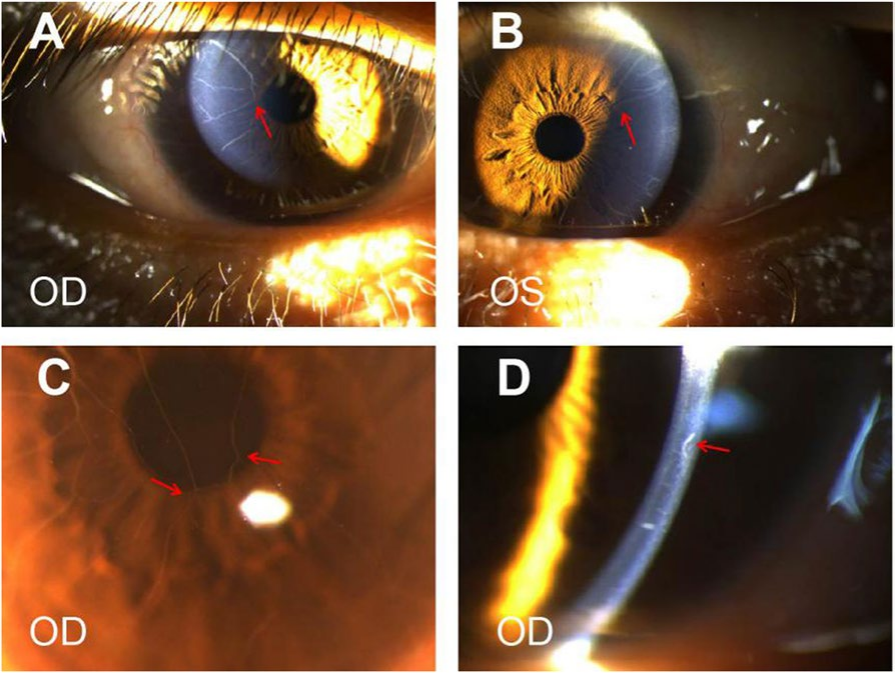
Corneal images. **a b** and **c** Sharply marginated 2–4 branches of markedly prominent corneal nerves extending onto the central cornea from corneoscleral limbus, partial interfitting (red arrow). The closer to the center of the cornea, the thinner the branch. **d** Prominent corneal nerves (magnification × 25)

A 41-year-old patient presented to the ophthalmology clinic with the complaint of itchy eyes and irritation, without blurred vision. The patient’s medical and surgical history was unremarkable. The patient’s uncorrected visual acuity was log MAR visual acuity 0.0 OD and log MAR visual acuity 0.0 OS, with normal ocular alignment. Slit-lamp examination in both eyes revealed mild obstruction of terminal duct and orifice in the upper and lower eyelids, light conjunctival hyperemia, and a semitransparent neoplasm measuring 2 mm*2 mm on the nasal corneal limbus (Fig. 1), without eversion and thickening of the upper or lower eyelid. During the examination, he was noted to have markedly prominent corneal nerves bilaterally in the anterior 2/3 part of the midcorneal stroma throughout the center and periphery of the cornea, varying in length (Fig. 2). In addition, a pink semitransparent neoplasm was seen on the inferior conjunctival fornix of the left eye (Fig. 1). The rest of the findings of the intraocular examination were unremarkable; intraocular pressure and tear film breakup time (BUT) were normal in both eyes. The patient also had thick lips, a premaxillary protrusion (overbite), and café au lait spots on the back of the left forearm.

**Fig. 3:**
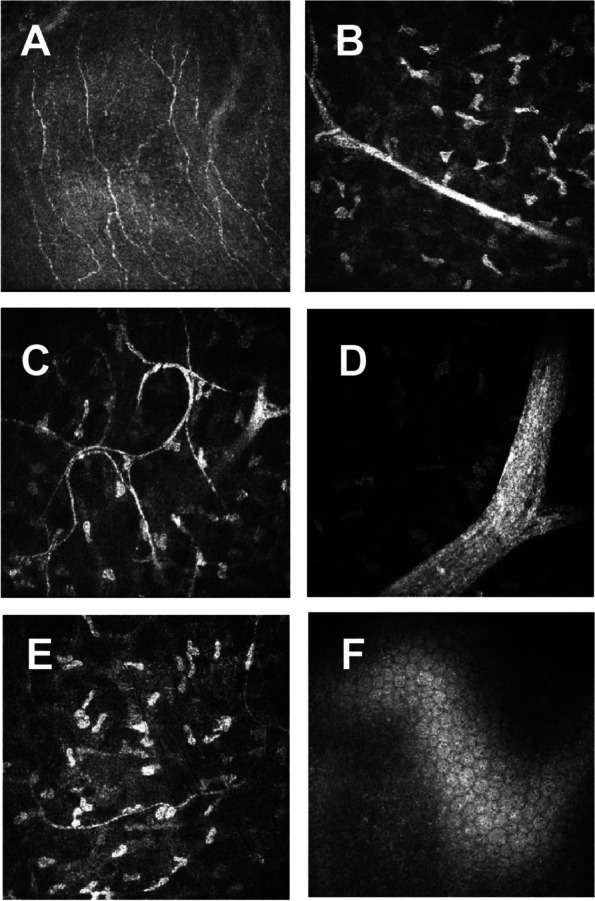
In vivo confocal microscopic images of cornea. No obvious abnormalities were found in the corneal pterygoid cells and basal cells. **a** Corneal sparse subbasal nerve plexus. **b** Hypertrophic nerves in anterior stroma. **c** A few thin nerve fibers forming loops and showing nodular dilatations. Thickened main corneal nerve trunk appearing as a bright, hyperreflective structure, Penetrating and bifurcating here. **d** Highly reflective hypertrophic nerves with bifurcations in midstroma, about 80 μm in diameter. **e** Thin nerve fibers present in posterior stroma. **f** Normal endothelium

**Fig. 4:**
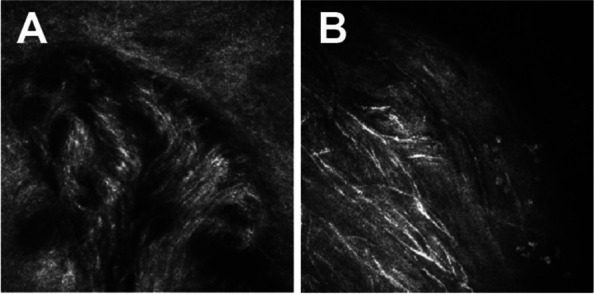
Conjunctival and paralimbal neuromas OS-1, 2, Slit-lamp images. **a** and **b** IVCM showing disorganized bundle of nerves (arrows) composed of elementary hyperreflective nerves with bifurcations, loops, and dilations

Pathological results of conjunctival semitransparent neoplasm could not be obtained because the patient refused to accept the surgical resection. However, in vivo confocal microscopy (IVCM) is a non-invasive method used to assess the living corneal nerve morphology in both physiological and pathological states. IVCM was performed on the cornea with the Heidelberg retinal tomograph III Rostock cornea module. Corneal IVCM images of the patient revealed a normal endothelium, with a sparse hyperreflective and thickened nerve plexus in both eyes (Fig. 3). IVCM images of neuromas showed a disorganized bundle of nerves composed of elementary hyperreflective nerves with bifurcations, loops, and dilations (Fig. 4).

The patient was referred to the endocrinology department for further screening for associated neoplasia. There was no known family history of endocrine malignancy. Physical examination showed a healthy man without enlarged thyroid glands or neck masses. However, the bilateral cervical lymph nodes were slightly enlarged. Hematologic examination showed that calcitonin was 15.14 pg/ml (normal value < 9.52 pg/ml). Ultrasound images showed uneven density/echoes in the thyroid. Cranial CT and MRI revealed no abnormal findings. Genetic testing identified a heterozygous SOS1 gene mutation (c.3263dup) instead of a mutation in RET gene. Therefore, multiple endocrine neoplasia (MEN) type 2B was preliminarily ruled out by an endocrinologist.

The patient was treated with topical 0.1% fluorometholone eye drops 4 times a day and sodium hyaluronate eye drops 4 times a day.

## Discussion and conclusion

We present a 41-year-old male with mild symptoms of ocular surface irritation and was noted to have strikingly prominent corneal nerves and conjunctival neuromas but no evidence of RET mutation. Based on his physical signs and genetic test result, a tentative diagnosis of pure mucosal neuroma syndrome was made.

Prominent corneal nerves can have a similar appearance to other phenomena. Ghost vessels are one such example that closely resemble corneal nerves on examination. Ghost vessels would have been larger and white. Lattice lines are another possibility; however, these lines are more opaque and overlap more than corneal nerves. Waite–Beckham lines could also present similarly, although these would be deep, vertical, and at the level of Descemet membrane.

Neurofibromatosis must be considered in any patient presenting with thickened and visible corneal nerves together with conjunctival neuromas. In vivo confocal microscopy analysis of NF1 showed morphological changes of the corneal structures; significant increases in the corneal endothelial cell density and in corneal nerve branching were observed, whereas corneal nerve density and the number of corneal nerve main trunks were not significantly different [13]. The IVCM of this patient, however, shows a prominent hyperreflective, thickened nerve plexus and a normal endothelium in our case, which differ from the increased corneal endothelial cell density and corneal nerve branching in an IVCM of NF1. Besides, this patient only displayed signs of three neurofibromas, meeting one of the clinical diagnostic criteria. Moreover, NF1 mutation was not detected in this patient. Therefore, the diagnosis of NF1 was excluded.

Multiple endocrine neoplasia (MEN) syndrome is an autosomal dominant disorder with three clinical subtypes: MEN1, MEN2 (MEN2A and MEN2B), and MEN4. MEN2B, also called Gorlin syndrome, is caused by germline mutations in the RET proto-oncogene and is characterized by medullary thyroid carcinoma and multiple mucosal neuromas and pheochromocytomas, with variable expressivity of intestinal ganglioneuromas, ophthalmic abnormalities, mucosal neuromas, and maxillofacial and orthopedic changes. Varying ophthalmic manifestations include enlarged prominent corneal nerves due to axon and Schwann cell abundance [14, 15], eyelid and subconjunctival neuromas, dry eye disease, lid margin eversion or thickening, ptosis, and prominent perilimbal blood vessels [16–18]. An IVCM of the cornea indicated an increased density and diameter of the subbasal nerve plexus and a normal-appearing endothelium [19]. The definitive diagnosis of MEN2 is made by RET sequencing [20]. In our case, multiple endocrine neoplasia (MEN) type 2B was preliminarily ruled out by an endocrinologist because there was no evidence of mutation in the RET proto-oncogene.

Pure mucosal neuroma syndrome (MSN) is a rare distinct subgroup of MEN2B, which presents with typical features of MEN2B but no evidence of mutation in the RET proto-oncogene or associated endocrinopathies (medullary thyroid carcinoma, pheochromocytoma). Prominent corneal nerves combined with conjunctival neuromas may be an early sign of MEN2B [17, 21]. Our patient had MEN2B-like ocular findings, but without a RET-gene mutation, medullary thyroid carcinoma, or pheochromocytoma, suggesting that he may represent a distinct subgroup termed “pure mucosal neuroma syndrome” [22]. Endocrinologist agreed with our diagnosis that they may have overlooked this rare distinct subgroup of MEN2B. A British investigation identified a genetic cause of pure MNS in which SOS1 frameshift mutations were related to isolated mucosal neuromas and gingival hypertrophy [23, 24]. In our case, testing for SOS1 mutation was positive, which is consistent with this British investigation [23, 24]. Nevertheless, a larger series of pure MNS patients is needed to confirm a detailed phenotype of this disorder. MEN2 patients do not require prophylactic thyroidectomy if they do not have RET mutation or biochemical evidence of C-cell hyperplasia [25]. Also, prophylactic thyroidectomy is not mandatory in these patients with MNS because patients with this important diagnosis have no recognized risk of early onset medullary thyroid cancer or pheochromocytoma [26].

In this report, the clinical and microstructural features of ocular involvement in NF1 and MEN2B are described. A detailed ocular examination and IVCM were performed in this patient. Ocular findings included mild obstruction of terminal duct and orifice in the upper and lower eyelids, light conjunctival hyperemia, conjunctival neuromas, and prominent corneal nerves. We detected a prominent hyperreflective and thickened nerve plexus and a normal endothelium in the IVCM for our case. Also, a heterozygous exon 20 mutation c.3263dup was found in the SOS1 gene. Our case illustrates the importance of recognizing the ocular features of MNS, a rare presentation of MEN2B, which is autosomal dominant and has no noted increased risk of early onset medullary thyroid cancer or pheochromocytoma. However, it is essential to follow up and screen regularly all patients with suspected MEN2B regardless of genotype.

In conclusion, differential diagnosis is required in all patients with prominent corneal nerves. Team work with endocrinologists and geneticists is also needed when patients presented with prominent corneal nerves and conjunctival neuromas to determine the true diagnosis and set the treatments appropriately.

## Data Availability

The datasets used during the current study are available from the corresponding author on reasonable request.

## Abbreviations

MEN: Multiple endocrine neoplasia
IVCM: In vivo confocal microscopy
MNS: Pure mucosal neuroma syndrome
BUT: Tear film breakup time
NF: Neurofibromatosis
